# Single-cell analysis reveals bronchoalveolar epithelial dysfunction in COVID-19 patients

**DOI:** 10.1007/s13238-020-00752-4

**Published:** 2020-07-15

**Authors:** Jiangping He, Shuijiang Cai, Huijian Feng, Baomei Cai, Lihui Lin, Yuanbang Mai, Yinqiang Fan, Airu Zhu, Huang Huang, Junjie Shi, Dingxin Li, Yuanjie Wei, Yueping Li, Yingying Zhao, Yuejun Pan, He Liu, Xiaoneng Mo, Xi He, Shangtao Cao, FengYu Hu, Jincun Zhao, Jie Wang, Nanshan Zhong, Xinwen Chen, Xilong Deng, Jiekai Chen

**Affiliations:** 1 Guangzhou Regenerative Medicine and Health-Guangdong Laboratory (GRMH-GDL), Guangzhou Institutes of Biomedicine and Health, Chinese Academy of Sciences, Guangzhou, 510530 China; 2 Guangzhou Eighth People’s Hospital, Guangzhou Medical University, Guangzhou, 510060 China; 3 The Centre of Cell Lineage and Atlas (CCLA), Guangzhou Regenerative Medicine and Health-Guangdong Laboratory, Guangzhou, 510530 China; 4 State Key Laboratory of Respiratory Disease, National Clinical Research Center for Respiratory Disease, Guangzhou Institute of Respiratory Health, The First Affiliated Hospital of Guangzhou Medical University, Guangzhou, 510120 China; 5 Key Laboratory of Regenerative Biology of the Chinese Academy of Sciences and Guangdong Provincial Key Laboratory of Stem Cell and Regenerative Medicine, Guangzhou Institutes of Biomedicine and Health, Chinese Academy of Sciences, Guangzhou, 510530 China; 6 University of the Chinese Academy of Sciences, Beijing, 100049 China

**Dear Editor,**

In 2019, a zoonotic coronavirus named severe acute respiratory syndrome coronavirus 2 (SARS-CoV-2) was identified as the causative agent of Coronavirus Disease 2019 (COVID-19). As of 8 June 2020, the World Health Organization (WHO) has reported 6,912,751 globally confirmed cases with 400,469 deaths.

Although generally causes mild disease, SARS-CoV-2 infection can result in serious outcomes, including acute lung injury (ALI) and acute respiratory distress syndrome (ARDS), the leading cause of mortality in patients with comorbidities. Recent autopsy studies of COVID-19 patients revealed mononuclear infiltration and excessive production of mucus in the infected lung, especially in the damaged small airways and alveoli (Bian and Team, 2020; Liu et al., 2020). This finding is a critical warning for clinical treatment, as if the mucus accumulation not dissolved, oxygen alone may not be able to achieve the goal for necessary breathing. Currently, no vaccines or specific anti-viral drugs have been approved for SARS-CoV-2, meriting the urgent development of novel therapies.

Thick mucus from coughs is a major clinical and pathological feature of COVID-19 and one of the most frequent symptoms in severe cases. Mucus plays a vital role in protecting the lungs against inhaled environmental injurious substances. The mucus-entrapped foreign materials are necessarily removed from the airways via the aid of coordinated ciliary beating or mucociliary clearance (MCC) (Fahy and Dickey, 2010). Conversely, in muco-obstructive airway disease, mucus becomes pathologic. Mucin hypersecretion and altered mucin macromolecular organization form a dysfunctional mucus gel (Fahy and Dickey, 2010). As a consequence, mucus accumulated in airways obstructs the respiratory tract lumen and limits airflow. Mucus accumulation can also cause by ciliary dysfunction, which has been observed in many diseases, including primary ciliary dyskinesia (PCD), cystic fibrosis (CF), asthma, chronic obstructive pulmonary disease (COPD), SARS-CoV, MERS-CoV and influenza virus infection (Tilley et al., 2015; Bustamante-Marin and Ostrowski, 2017; Fong, 2017; Chen et al., 2019).

At present, the disease pathogenesis of mucus hypersecretion leads ARDS in COVID-19 remains largely unknown. Here, we report the leukocytes and epithelial cells in the bronchoalveolar lavage fluid (BALF) from 3 severe COVID-19 patients (Table S1) at single-cell resolution and investigate the SARS-CoV-2 infection-induced ARDS at the cellular and molecular levels.

We performed single-cell RNA sequencing (scRNA-seq) based on the 10X genomics platform, passing through a rigorous quality-control process, the transcriptome profiles of 8,356 cells were subjected to downstream analysis. Firstly, we repurposed and integrated a public pulmonary scRNA-seq dataset (Reyfman et al., 2019) from 8 healthy donors as control, denoted HC Most cell types were consistent among the COVID-19 and HC samples, including macrophages, B cells, endothelial cells, ciliated cells, club cells, as well as type1 and type2 alveolar cells (AT1 and AT2) (Figs. 1A, 1B and S1A). Then we examined the involved cell type of SARS-COV-2 infection, we detected 23 cells with at least 2 viral mRNA reads (Fig. S1B), but containing minimal numbers of expressed genes (Fig. S1C), indicating that SARS-CoV-2 suppresses host gene expression. It is difficult to define the cell types according to such low coverage of expressed genes. Thus, we tested self-organizing map (SOM), linear support vector machine (SVM), and random forest (RF) for cell type prediction, RF perform best in our test data with the known cell identities (Fig. S1D). Thus, we build an RF based classifier (Fig. S1E) and unveiled monocytes/neutrophils and club cells as potential viral targets (Fig. S1F).

**Figure 1:**
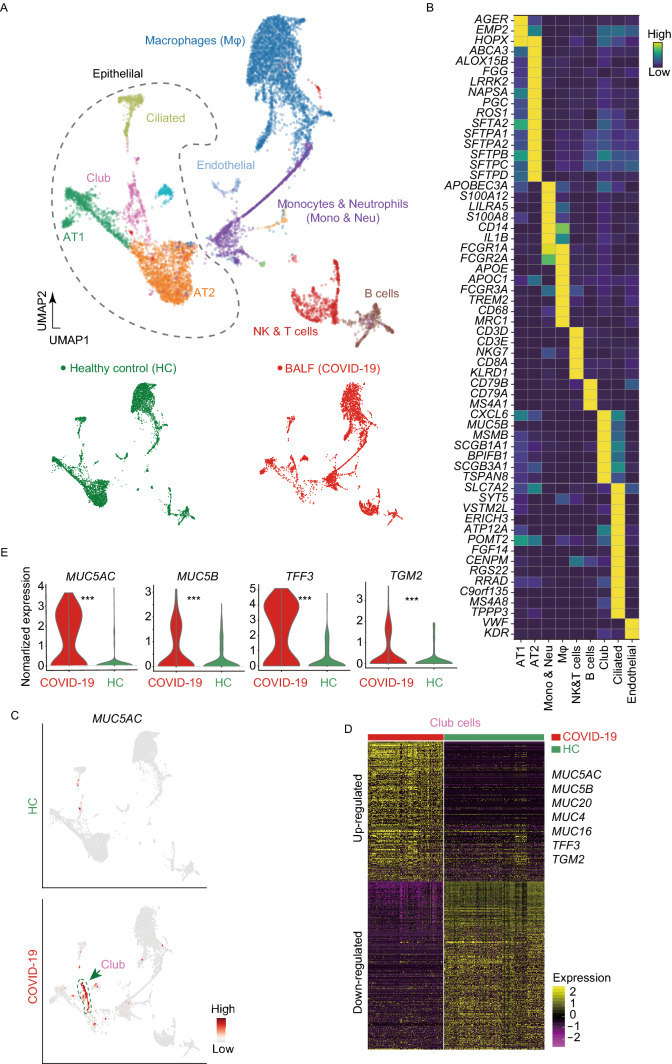

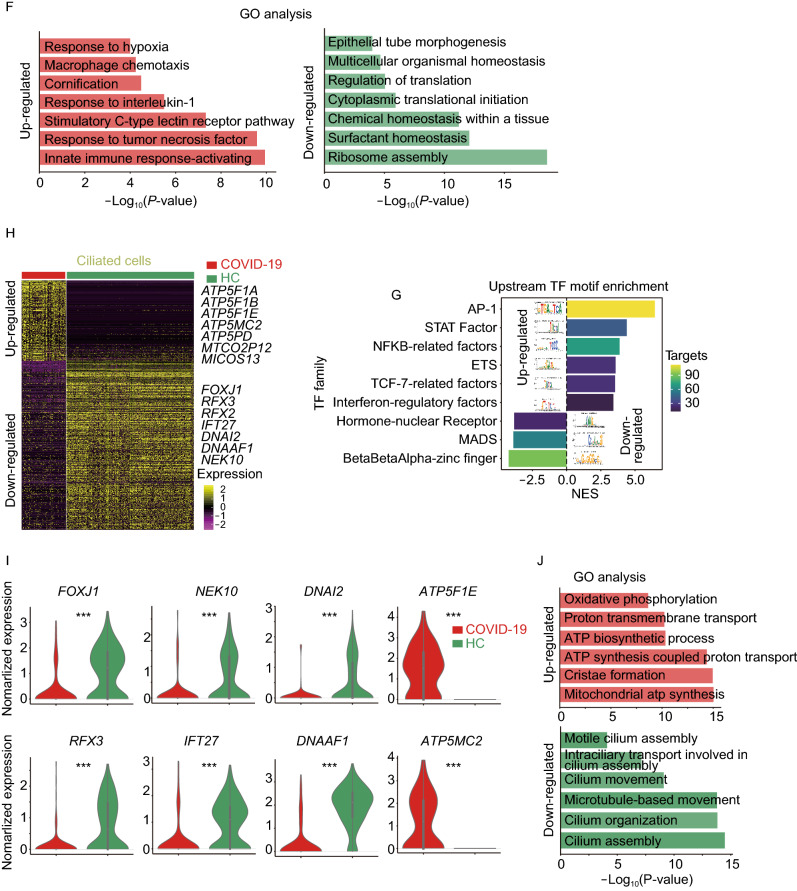
**Mucin hypersecretion from Club cells upon SARS-CoV-2 infection**. (A) (Upper) UMAP plots visualizing single-cell RNA-seq data of 4,237 BALF single cells from COVID-19 patients and 6,100 lung single cells from 8 healthy control (HC). (Lower) Differences in cellular composition among HC (green, left) and BALF (red, right). (B) Heatmap showing the average expression levels per cluster of the differentially expressed markers in each cluster. (C) UMAP depicting the expression of MUC5AC in HC and COVID-19 patients. (D) Heatmap depicting the differential expressed genes between HC and COVID-19 patients in Club cells. Selected genes are labeled in the right. All genes are significant differential expressed with fold change >1.5 and *P*-value < 0.01; *P*-value was from the Wilcoxon test. (E) Violin plots showing the differential expression of MUC genes between HC and COVID-19 patients. *** represents *P*-value < 0.001, *P*-value was from Wilcoxon test. (F) Gene ontology (GO) analysis for the differential expressed genes from panel (D). (G) Motif and TF enrichment for the differentially expressed genes from panel (D). The motif ranking database hg38__refseq-r80__10kb_up_and_down_tss was used for enrichment and motifs were group by the family of the corresponding transcription factors. (H) Heatmap depicting the differential expressed genes in Ciliated cells between HC and COVID-19 patients. (I) Violin plots showing the expression of cilium formation related genes between HC and COVID-19 patients. (J) GO analysis for the differential expressed genes from panel (H)

Next, we compared the differential expressing genes (DEGs) of each cell type between HC and COVID-19. MUC5AC is the major component of secreted mucins in the airways (Lillehoj et al., 2013; Krishn et al., 2018). Our analysis revealed a significant elevation of *MUC5AC* expression in patients, specifically in the club cells (Fig. 1C), suggesting club cells are predominant for mucins secretion post-infection. Our analysis revealed upregulation of additional mucin genes including the gel-forming mucins (GFM), *MUC5AC* and *MUC5B,* as well as membrane-tethered mucins, *MUC4*, *MUC16* and *MUC20* (Lillehoj et al., 2013) in patients’ club cells (Fig. 1D and 1E), and these observations were consistent across all patients (Fig. S1G). Meanwhile, *TFF3* and *TGM2*, which can mediate cross-links between GFM fibers to form supramolecular assembly by isopeptide bonds, which will increase mucus viscosity and impairs MCC (Demouveaux et al., 2018), were also significantly increased in COVID-19 patients (Fig. 1D and 1E). Gene ontology (GO) analysis further demonstrated the COVID-19-specific enrichment of stimulatory C-type lectin receptor, response to IL-1 and TNF, hypoxia, as well as macrophage chemotaxis (Fig. 1F). Specifically, IL-1β and TNF-α were known to induce mucin production in surface epithelial cells (Krishn et al., 2018), illustrating the involvement of immune regulators. In contrast, the homeostasis and epithelial tube morphogenesis related genes were downregulated (Figs. 1F and S1H), suggesting lung damage in COVID-19. To understand the molecular basis, we enriched potential upstream transcription factors (TF) based on the DEGs of club cells, and results revealed 6 potential TF families were enriched in COVID-19 patients (Fig. 1G), including activator protein-1 (AP-1) and NFKB, which are known involved in IL-1β and TNF-α induced MUC5AC promoter activation respectively (Krishn et al., 2018). Taken together, these results demonstrated that COVID-19-stimulated mucin secretion in the club cells, potentially through the innate immune regulators, IL-1β and TNF-α, and leading to ARDS.

Furthermore, our results unveiled the master regulators for cilia generation, including *FOXJ1* and *RFX3* (Bustamante-Marin and Ostrowski, 2017), which were significantly down-regulated upon viral infection (Figs. 1H, 1I, and S2A). The expression of *NEK10*, the cilia-specific kinase whose activity promotes ciliary length and mucociliary transport (Chivukula et al., 2020), also dropped (Figs. 1H, 1I, and S2A). Besides, we noted the dampened transcription of ciliary structural (i.e., *DNAI2, DNAF1, SPAG16, INTU, and AHI*) and functional (i.e., *IFT27*) genes (Figs. 1H, 1I and S2A) (Bustamante-Marin and Ostrowski, 2017). Conversely, the ATP syntheses (e.g., *ATP5F1E, ATP5MC2,* and *ATP5MG*) were dramatically increased in COVID-19 patients (Figs. 1I and S2B). It is worth noting that, upon stimulation by high levels of extracellular ATP, the coordinate ciliary beat frequency disrupt and airway mucin secretion can be increased thousands-fold (Fahy and Dickey, 2010; Bustamante-Marin and Ostrowski, 2017). GO analysis again highlighted reduced ciliary maintenance and increased ATP synthesis pathways upon infection (Fig. 1J). Above all, these data demonstrated ciliary dysfunction post-SARS-CoV-2 infection.

Next, we assessed the contribution of AT1 and AT2 cells in COVID-19. The keratinization-related genes, including *CSTA* and *KRT13,* were significantly up-regulated (Fig. S2C–F). While four critical surfactant proteins (SPs)—SP-A, SP-B, SP-C, and SP-D, known to maintain the structural integrity of alveoli and facilitate gas exchange, were dramatically down-regulated in COVID-19 disease (Fig. S2C–E and S2G). Besides, the level of *NKX2-1*, the transcription factor required for surfactant synthesis was also reduced (Fig. S2H), indicating the loss of alveoli integrity and the possible pathogenesis of respiratory distress syndrome in COVID-19.

To evaluate the impact of the immune microenvironment on epithelial dysfunction upon viral infection, we dissected the transcriptomic profiles of three major regulators of innate immunity, monocytes, neutrophils, and macrophages. Increased levels of generalized inflammatory chemokines, including *CCL2*, *CXCL8*, *CCL3*, *CCL4*, *CXCL2*, *CCL3L1*, *IL1RN*, and *IL1B*, as well as the anti-viral restriction factor, *IFITM3*, were observed amongst the COVID-19 patients (Figs. 2A–E, S3A and S3B), reflecting exacerbated inflammatory response post-infection. Moreover, CCL2 was specifically elevated in the patients’ macrophages (Fig. 2E), possibly inducing the recruitment of inflammatory monocytes/neutrophils to the infection sites (Qian et al., 2011). Consistently, GO analysis demonstrated the enrichment of interferon and interleukin-associated pathways in the COVID-19-infected monocytes and neutrophils (Fig. S3C), highlighting the escalated innate immune defense upon infection. In the study of Liao et al. (2020), proinflammatory macrophages expressed *FCN1* were found abundant in BALF from patients with severe COVID-19. However, we noticed only a mild increase of *FCN1*+ macrophages but significantly decreased *FCN1*+ monocytes/neutrophils in COVID-19 patients (Figs. 2F and S3D), indicating the distinct immune profiles among COVID-19 patients.

**Figure 2:**
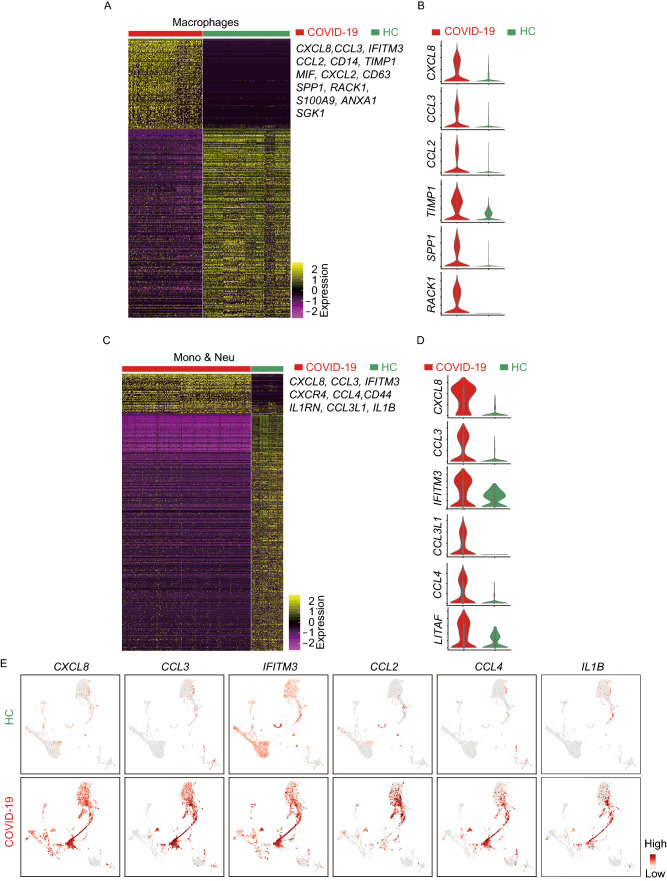

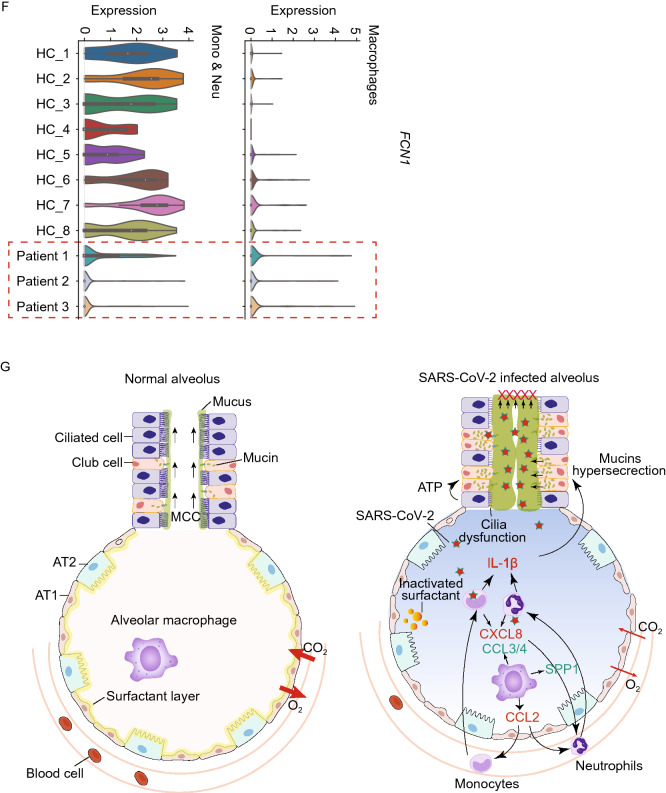
**Differential expressing genes reveals macrophages and monocytes/neutrophils anti-viral response**. (A) Heatmap showing the differential expressed genes between HC and COVID-19 patients in macrophages, selected genes are indicated in the right. (B) Violin plots depicting the selected differential expressed genes between HC and COVID-19 patients in macrophages. (C) Heatmap showing the differential expressed genes between HC and COVID-19 patients in monocytes/neutrophils. (D) Violin plots depicting the selected differential expressed genes between HC and COVID-19 patients in monocytes/neutrophils. (E) UMAP showing the expression of selected genes between HC and COVID-19 patients. (F) Violin plots showing the expression of *FCN1* in macrophages (upper) or monocytes/neutrophils (lower). (G) Schematic diagram of the normal alveolus (left) and the severe COVID-19 alveolus (right)

To further scrutinize the immune features of the innate immune players, we depicted the cell fate trajectory of monocyte, neutrophil, and macrophage subtypes (Fig. S3E). Notably, MIP-1 chemokines, CCL3 (MIP-1α) and CCL4 (MIP-1β), as well as MIP-1-induced proinflammatory cytokines, IL-1β and TNF distributed among monocyte and neutrophil subgroup 2 cells (Fig. S3E–G), demonstrating their potency in the inflammatory response as well as the potential impairment of airway epithelial function and MCC.

To investigate the consequence of monocyte and neutrophil activation, we analyzed the differentially expressed genes of the NK and T cell populations in both healthy and COVID-19 individuals (Fig. S3H). Notably, antigen processing pathways were up-regulated (Fig. S3I), whereas lost *IL-7R* and *IL-2* expressions (Fig. S3J), indicating the hindered T cell survival.

SARS-CoV-2, the causative pathogen of COVID-19 pneumonia, has spread worldwide. The clinical symptoms of COVID-19 vary from asymptomatic to severe ARDS. Currently, no approved anti-viral drug, vaccine, or effective clinical treatment for COVID-19 due to the lack of understanding of disease pathogenies. Here we comprehensively dissected the epithelial and immune profiles of BALF derived from severe COVID-19 patients at a single-cell level. Specifically, monocytes/neutrophils driven pulmonary epithelial dysfunction upon viral infection was characterized in molecular details, revealing high-inflammation and mucin hypersecretion of the infected respiratory tract. The exacerbated inflammatory response was observed post-infection, featured by the drastic elevation of IL-1β, the activators of mucin production. Further, mucin hypersecretion was primarily detected in the club cells of the patients, highlighting the major source of mucus in COVID-19 disease. Besides, deficiency of ciliary structural components and dysregulation of ATP production were uncovered in the patients’ cilia, demonstrating ciliary dysfunction and impaired mucus removal machinery. In turn, mucus build-up would disseminate infection and amplify inflammation, leading to ALI and ARDS (Fig. 2G). Overall, our results revealed monocytes/neutrophils-released pro-inflammation cytokines, IL-1β as the major driving force towards airway epithelial dysregulation and ARDS in severe COVID-19 patients.

Rapid onset of widespread inflammation in the lungs leads to ARDS and subsequent fatality in COVID-19. To reveal the mechanism bridging exacerbated immune response and ARDS, our study provided solid evidence from various perspectives of epithelial dysfunction, encompassing club cell-driven hyper-production of mucins, dysrhythmic ciliary movement, and compromised alveoli integrity. However, mucin hypersecretion and altered mucin macromolecular organization form a dysfunctional mucus gel, which is difficult to be removed by drugs or sputum aspirator, highlighting the clinical value of removing the mucins at the earlier stage. At the molecular level, our results revealed these malfunctioned biological processes had been tightly associated with IL-1β induced inflammatory microenvironment. Notably, recently a retrospective cohort study reported that treatment with high-dose anakinra, an IL-1 receptor antagonist, was safe and associated with clinical improvement in 72% COVID-19 patients (Cavalli et al., 2020). Highlighted blocking IL-1β holds significant translational value.

Liao et al. (2020) have surveyed the BALF immune response in COVID-19 patients and only focused on the immune cells like macrophage and CD8^+^ T cells. In contrast, our study takes a significant analysis of lung epithelial cells. It reveals mucin hypersecretion and ciliary dysfunction in severe COVID-19 patients, which would lead to acute lung injury and ARDS. Furthermore, our study revealed the highly activated monocytes and neutrophils, instead of macrophages, were the source of the exacerbated immune response characterized by increased IL1β.

Taken together, our investigation provides pathological and mechanistic insights of ARDS in severe COVID-19 patients and provided translational insights towards the treatment of severe COVID-19 cases.

## Electronic supplementary material

Below is the link to the electronic supplementary material.

Supplementary material 1 (PDF 22896 kb)

Supplementary material 1 (XLS 19 kb)
