# Supplementary material for: Single-cell analysis reveals bronchoalveolar epithelial dysfunction in COVID-19 patients
Source: Protein Cell. 2020 Jul 15;11(9):680–7. doi: 10.1007/s13238-020-00752-4 (PMC7363016; doi:10.1007/s13238-020-00752-4)
Supplement: 13238_2020_752_MOESM1_ESM — MATERIALS AND METHODS [file 13238_2020_752_moesm1_esm.pdf]

MATERIALS AND METHODS

BALF sample collection

BALF samples collection was conducted by professional nurses in the hospital and were processed in a biosafety level 2 plus laboratory with biosafety level 3 personal protection equipment. Cells in the BALF were freshly used for 10x single-cell RNA-seq.

Single-Cell RNA library preparation and sequencing

Cell suspensions were loaded onto a chromium single-cell chip to generate single-cell gel bead-in-emulsions (GEMs) aiming for 2,000–8,000 single cells per reaction. The single-cell 3'-library was constructed using Chromium Single Cell Reagent Kits v3 (10X GENOMICS) following the manufacturer's user guide. Following cell lysis, first-strand cDNA synthesis and amplification were carried out according to the instructions. Amplified cDNA was purified using SPRIselect beads (Beckman Coulter) and sheared to 250-400 bp. cDNA quality control was performed using Qubit 3.0 Fluorometer and Agilent Bioanalyzer 2100. The linear DNA libraries were converted to a single-stranded circular(ssCir) DNA library by MGI Easy Universal Library Conversion Kit (App-A, MGI) and sequenced on BGISEQ-500 with high-throughput sequencing set (App-A, MGI) with the following read lengths: 28-bp read 1 (containing the 18-bp cell barcode and 10-bp randomer), 100-bp read 2 and 8-bp barcodes.

Single-cell RNA-seq data analysis

scRNA-seq data was processed using the scTE (https://github.com/jphe/scTE) 10x pipeline, Briefly, reads were aligned to the human genome (hg38) using STARsolo(Dobin et al., 2013) with the setting '--outSAMattributes NH HI AS nM CR CY UR UY --readFilesCommand zcat --outFilterMultimapNmax 100 --winAnchorMultimapNmax 100 --outMultimapperOrder Random --runRNGseed 777 --outSAMmultNmax 1'. The default scTE parameters for 10x were used to get the molecule count matrix. The count matrix was lightly filtered to exclude cell barcodes

with low numbers of counts: Cells with less than 2000 UMIs, less than 300 genes detected or more than 40% fraction of mitochondrial counts were removed. For comparison between COVID-19 patient BALF and healthy control lung, the batch effect was corrected by Seurat (V3)(Stuart et al., 2019). The genes with fold change >1.5 and adjusted P-value < 0.01 (Wilcoxon test) were considered to be differentially expressed. The Gene Ontology (GO) analysis was performed by clusterProfiler (Yu et al., 2012). Transcription factor motif enrichment was performed by RcisTarget(Aibar et al., 2017). Other analysis was performed by SCANPY (Wolf et al., 2018).

SARS-CoV-2 infected cell type prediction

The labeled COVID-19 BALF and Health Control data were used as training set. We calculated the expression rate of each gene with UMI > 0 in infected cells and selected the top 1000 genes. In addition, the differential expressed genes between HC and COVID-19 patients were excluded to avoid bias relevant to pathological state. These procedures resulting 689 genes, which were used for cell type classification and prediction. The logarithmic gene expression values were used as input. To find a suitable classifier, we tested Self-organizing map (SOM), linear Support Vector Machine (SVM) and Random Forest (RF). In detail, linear kernel with C=1 for SVM implemented in R package e1071, where C is the constant of the regularization term. 15×15 hexagonal grid for SOM implemented in R package kohonen. ntree=500 and mtry=159 for RF implemented in R package randomForest. 10-fold cross validation implemented in R package caret was used to assess the performance in training set. The average area under receiver operating characteristic curve (AUC) was used as model accuracy metric. RF is the best algorithm in this scene.

REFERENCES

Aibar, S., Gonzalez-Blas, C.B., Moerman, T., Huynh-Thu, V.A., Imrichova, H., Hulselmans, G., Rambow, F., Marine, J.C., Geurts, P., Aerts, J., *et al.* (2017). SCENIC: single-cell regulatory network inference and clustering. Nat Methods 14, 1083-1086.

Dobin, A., Davis, C.A., Schlesinger, F., Drenkow, J., Zaleski, C., Jha, S., Batut, P., Chaisson, M., and

Gingeras, T.R. (2013). STAR: ultrafast universal RNA-seq aligner. Bioinformatics 29, 15-21.
Stuart, T., Butler, A., Hoffman, P., Hafemeister, C., Papalexi, E., Mauck, W.M., 3rd, Hao, Y., Stoeckius, M., Smibert, P., and Satija, R. (2019). Comprehensive Integration of Single-Cell Data. Cell 177, 1888-1902 e1821.
Wolf, F.A., Angerer, P., and Theis, F.J. (2018). SCANPY: large-scale single-cell gene expression data analysis. Genome Biol 19, 15.
Yu, G., Wang, L.-G., Han, Y., and He, Q.-Y. (2012). clusterProfiler: an R package for comparing biological themes among gene clusters. Omics : a journal of integrative biology 16, 284-287.

Figure S1. Dysregulated transcriptome profile of Club cells in COVID-19 patients.

(A) UMAP showing the expression of top differential expressed marker genes.

(B) Violin plot shows the raw UMI counts of uninfected and infected cells. The cells in which more than two viral UMIs were detected were defined as infected cells (n=23).

(C) Boxplot depicting the detectable expressed genes in uninfected and infected cell cluster.

(D) Boxplot showing the the average area under receiver operating characteristic curve (AUC) representing the prediction accuracy of different method for the test-dataset which the cell identity was already known.

(E) Schematic plot depicting predicting the SARS-CoV-2 infected cell types using RF method, and both COVID-19 BALF cells and HC lung cells use as the annotation.

(F) Bar chart showing predicted cell types of the SARS-CoV-2 infected cells.

(G) Violin plots for the expression of MUC genes in club cells between different donors.

(H) Violin plots for the indicated genes in club cells between different donors.

Figure S2 Virus-induced response of bronchoalveolar epithelia cells in COVID-19 patients.

(A) Violin plots showing the expression of cilium formation related genes between different donors.

(B) Violin plots showing the expression of ATP synthesis related genes between different donors.

(C) Heatmap showing the differential expressed genes between HC and COVID-19 patients in AT1 cells.

(D) Heatmap showing the differential expressed genes between HC and COVID-19 patients in AT2 cells.

(E) GO analysis for the genes from panel C and D.

(F) Violin plots showing the expression of keratinization related genes between HC and COVID-19 patients in AT1 and AT2 cells.

(G) Violin plots showing the expression of surfactant homeostasis related genes between HC and COVID-19 patients in AT1 and AT2 cells.

(H) Violin plots showing the expression of *NKX2-1* between HC and COVID-19 patients in AT1 and AT2 cells.

Figure S3. Unique immune state for COVID-19 diseased lung.

(A) Violin plots showing the expression of indicated genes in macrophages for different donors.

(B) Violin plots showing the expression of indicated genes in monocyte/neutrophils for different donors.

(C) GO analysis for the differential expressed genes in macrophages and monocytes/neutrophils between HC and COVID-19 patients.

(D) UMAP showing the expression of FCN1 between HC and COVID-19 patients.

(E) The force-directed layout presentation of monocytes, neutrophils and macrophages in COVID-19 patients. Diffusion map was performed to denoise the expression data based on 1000 highly variable genes and 20 PCs. Top 5 diffusion components were selected for layout.

(F) Heatmap showing the differential expressed genes between different cluster of cells from panel E.

(G) Same as panel E, but cells are colored by the expression of indicated genes

(H) Heatmap showing the differential expressed genes between HC and COVID-19 patients in NK & T cells.

(I) GO analysis for the genes from panel H.

(J) Violin plots depicting the expression change for the selected genes between HC and COVID-19 patients in NK & T cells.

Figure S1

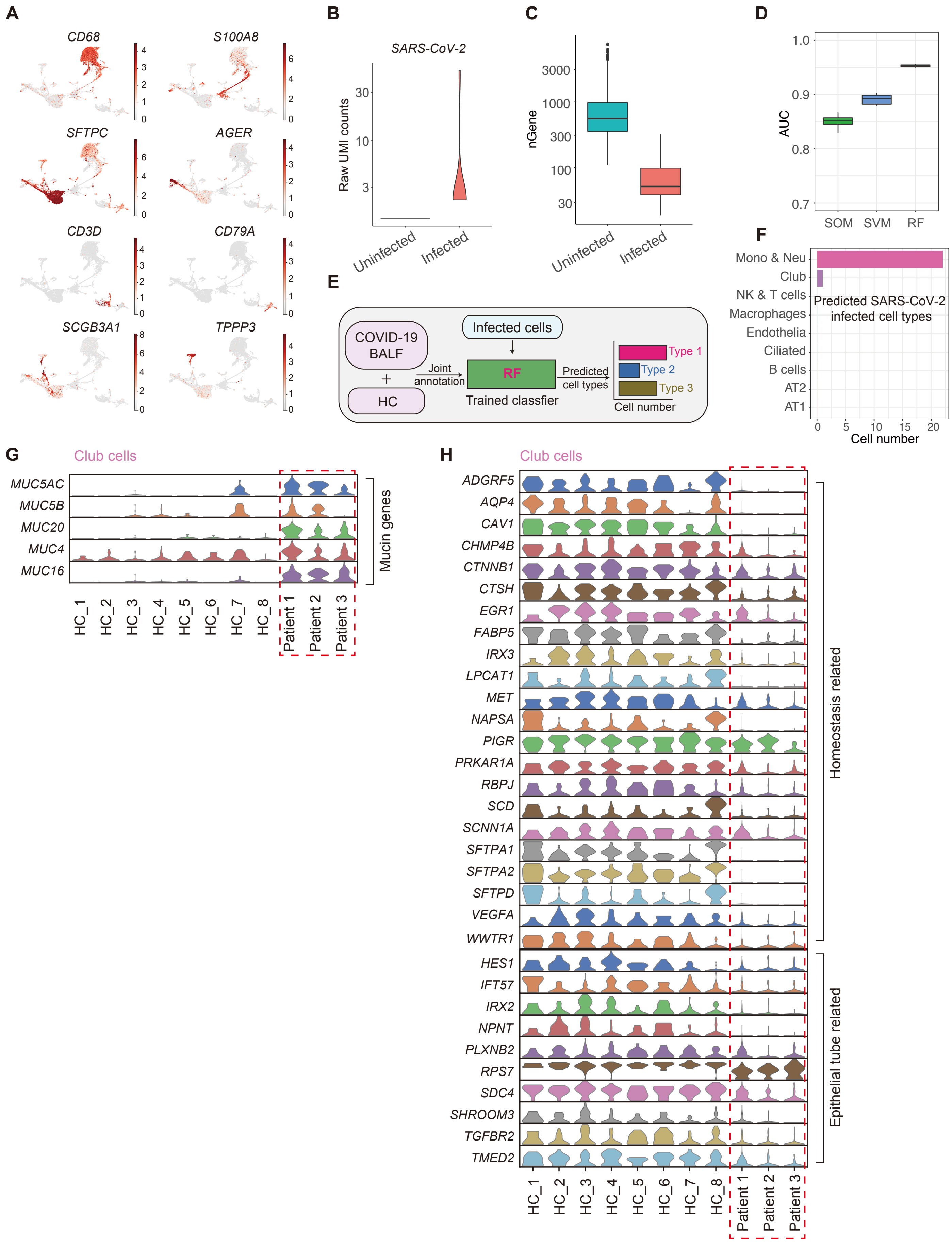

A
CD68
S100A8
SFTPC
AGER
CD3D
CD79A
SCGB3A1
TPPP3
B
SARS-CoV-2
Raw UMI counts
Uninfected
Infected
C
nGene
Uninfected
Infected
D
AUC
SOM
SVM
RF
E
COVID-19 BALF
HC
Joint annotation
Infected cells
RF
Trained classfier
Predicted cell types
Type 1
Type 2
Type 3
Cell number
F
Mono & Neu
Club
NK & T cells
Macrophages
Endothelia
Ciliated
B cells
AT2
AT1
Predicted SARS-CoV-2 infected cell types
Cell number
G
Club cells
MUC5AC
MUC5B
MUC20
MUC4
MUC16
Mucin genes
HC_1
HC_2
HC_3
HC_4
HC_5
HC_6
HC_7
HC_8
Patient 1
Patient 2
Patient 3
H
Club cells
ADGRF5
AQP4
CAV1
CHMP4B
CTNNB1
CTSH
EGR1
FABP5
IRX3
LPCAT1
MET
NAPSA
PIGR
PRKAR1A
RBPJ
SCD
SCNN1A
SFTPA1
SFTPA2
SFTPD
VEGFA
WWTR1
Homeostasis related
HES1
IFT57
IRX2
NPNT
PLXNB2
RPS7
SDC4
SHROOM3
TGFBR2
TMED2
Epithelial tube related

Figure S2

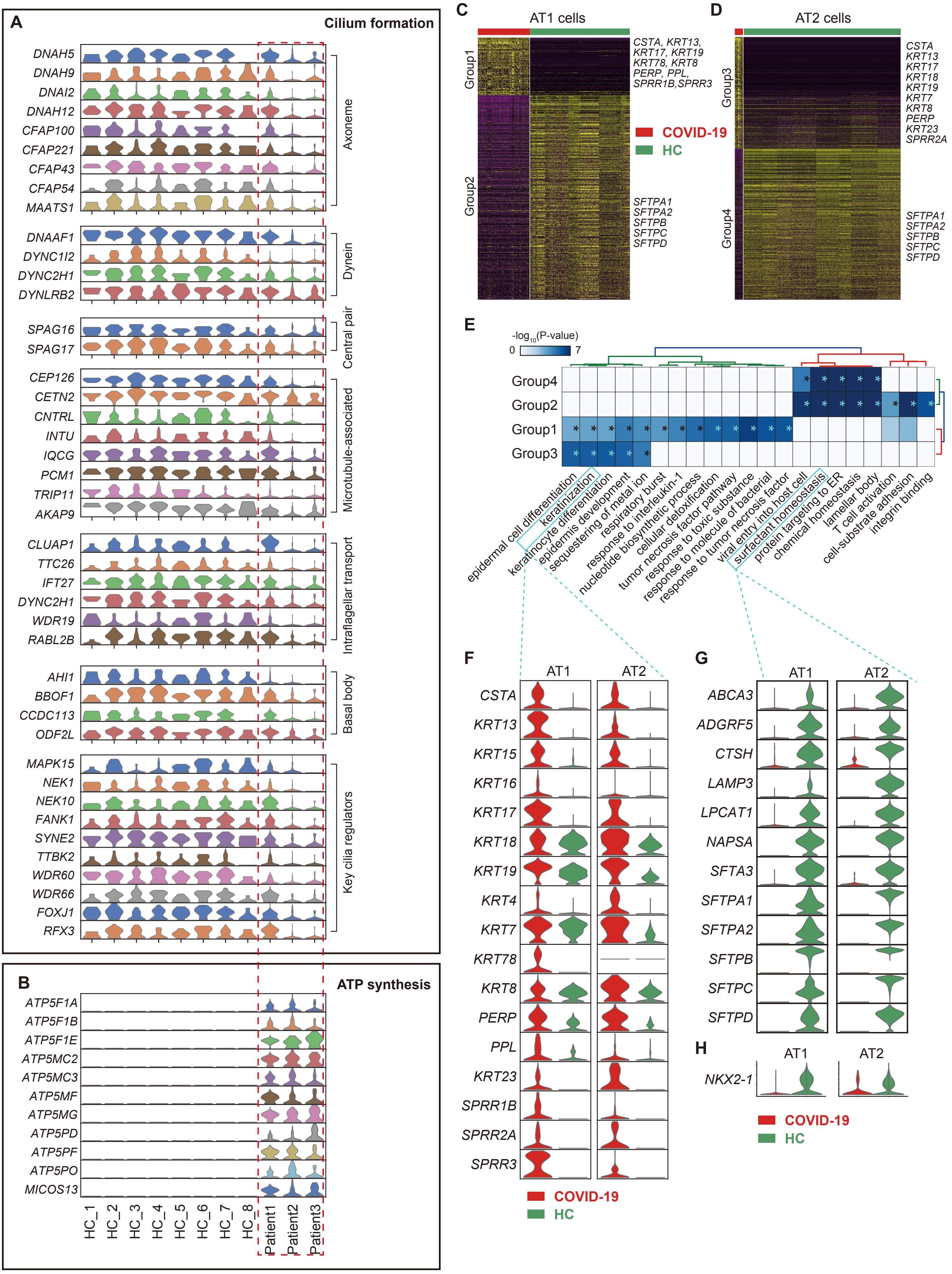
A
Cilium formation
DNAH5
DNAH9
DNAI2
DNAH12
CFAP100
CFAP221
CFAP43
CFAP54
MAATS1
Axoneme
DNAAF1
DYNC1I2
DYNC2H1
DYNLRB2
Dynein
SPAG16
SPAG17
Central pair
CEP126
CETN2
CNTRL
INTU
IQCG
PCM1
TRIP11
AKAP9
Microtubule-associated
CLUAP1
TTC26
IFT27
DYNC2H1
WDR19
RABL2B
Intraflagellar transport
AHI1
BBOF1
CCDC113
ODF2L
Basal body
MAPK15
NEK1
NEK10
FANK1
SYNE2
TTBK2
WDR60
WDR66
FOXJ1
RFX3
Key cilia regulators
B
ATP synthesis
ATP5F1A
ATP5F1B
ATP5F1E
ATP5MC2
ATP5MC3
ATP5MF
ATP5MG
ATP5PD
ATP5PF
ATP5PO
MICOS13
HC_1
HC_2
HC_3
HC_4
HC_5
HC_6
HC_7
HC_8
Patient1
Patient2
Patient3
C
AT1 cells
Group1
Group2
CSTA, KRT13,
KRT17, KRT19
KRT78, KRT8
PERP, PPL,
SPRR1B,SPRR3
SFTPA1
SFTPA2
SFTPB
SFTPC
SFTPD
COVID-19
HC
D
AT2 cells
Group3
Group4
CSTA
KRT13
KRT17
KRT18
KRT19
KRT7
KRT8
PERP
KRT23
SPRR2A
SFTPA1
SFTPA2
SFTPB
SFTPC
SFTPD
E
-log10(P-value)
0
7
Group4
Group2
Group1
Group3
epidermal cell differentiation
keratinization
keratinocyte differentiation
epidermis development
sequestering of metal ion
respiratory burst
response to interleukin-1
nucleotide biosynthetic process
cellular detoxification
tumor necrosis factor pathway
response to toxic substance
response to molecule of bacterial
response to tumor necrosis factor
viral entry into host cell
surfactant homeostasis
protein targeting to ER
chemical homeostasis
lamellar body
T cell activation
cell-substrate adhesion
integrin binding
F
AT1
AT2
CSTA
KRT13
KRT15
KRT16
KRT17
KRT18
KRT19
KRT4
KRT7
KRT78
KRT8
PERP
PPL
KRT23
SPRR1B
SPRR2A
SPRR3
COVID-19
HC
G
AT1
AT2
ABCA3
ADGRF5
CTSH
LAMP3
LPCAT1
NAPSA
SFTA3
SFTPA1
SFTPA2
SFTPB
SFTPC
SFTPD
H
AT1
AT2
NKX2-1
COVID-19
HC

Figure S3

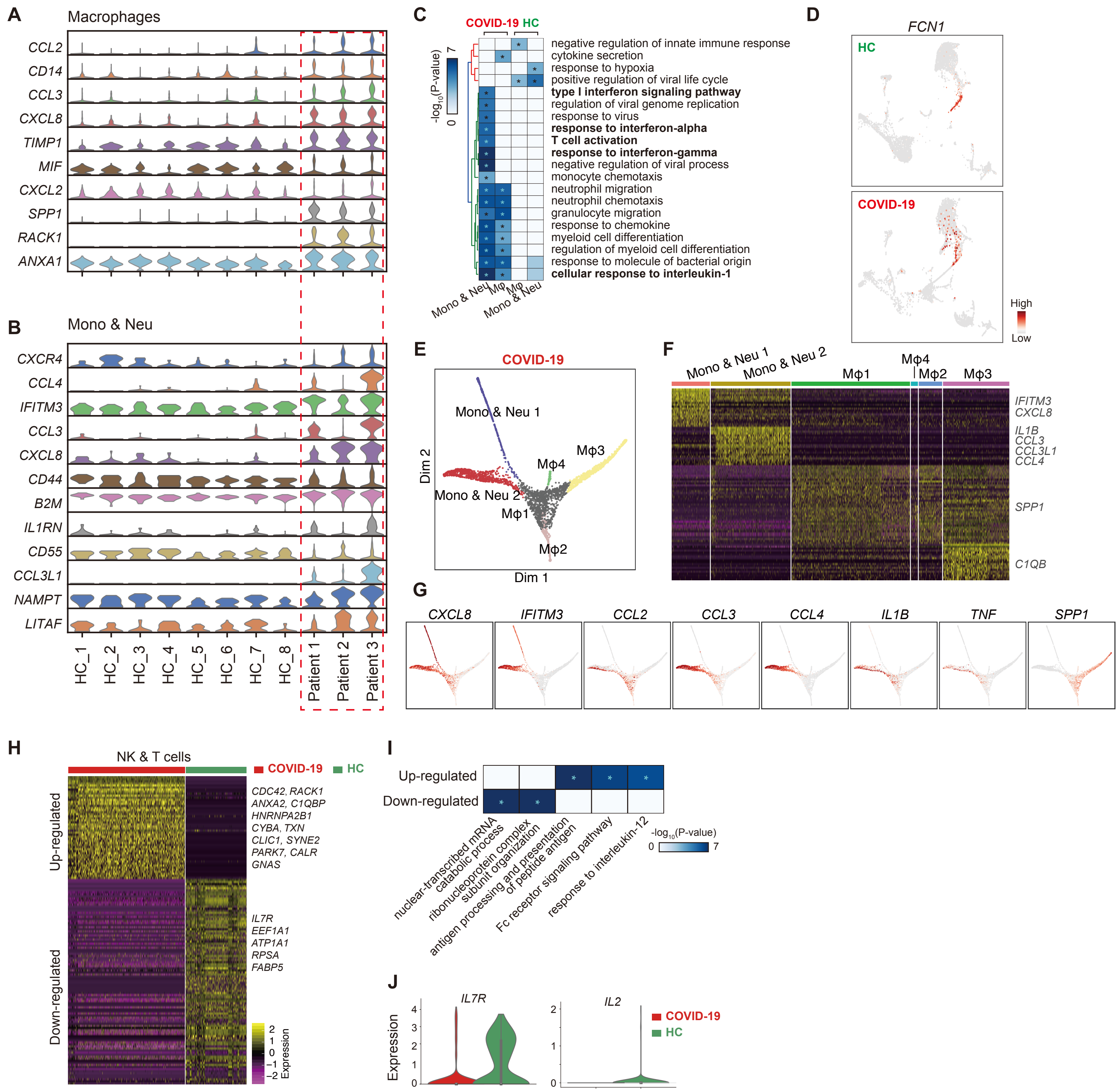

A
Macrophages
CCL2
CD14
CCL3
CXCL8
TIMP1
MIF
CXCL2
SPP1
RACK1
ANXA1
B
Mono & Neu
CXCR4
CCL4
IFITM3
CCL3
CXCL8
CD44
B2M
IL1RN
CD55
CCL3L1
NAMPT
LITAF
HC_1
HC_2
HC_3
HC_4
HC_5
HC_6
HC_7
HC_8
Patient 1
Patient 2
Patient 3
C
COVID-19
HC
-log10(P-value)
7
0
negative regulation of innate immune response
cytokine secretion
response to hypoxia
positive regulation of viral life cycle
type I interferon signaling pathway
regulation of viral genome replication
response to virus
response to interferon-alpha
T cell activation
response to interferon-gamma
negative regulation of viral process
monocyte chemotaxis
neutrophil migration
neutrophil chemotaxis
granulocyte migration
response to chemokine
myeloid cell differentiation
regulation of myeloid cell differentiation
response to molecule of bacterial origin
cellular response to interleukin-1
Mono & Neu
Mφ
Mφ
Mono & Neu
D
FCN1
HC
COVID-19
High
Low
E
COVID-19
Mono & Neu 1
Mφ3
Mφ4
Mono & Neu 2
Mφ1
Mφ2
Dim 2
Dim 1
F
Mono & Neu 1
Mono & Neu 2
Mφ1
Mφ4
Mφ2
Mφ3
IFITM3
CXCL8
IL1B
CCL3
CCL3L1
CCL4
SPP1
C1QB
G
CXCL8
IFITM3
CCL2
CCL3
CCL4
IL1B
TNF
SPP1
H
NK & T cells
COVID-19
HC
Up-regulated
Down-regulated
CDC42, RACK1
ANXA2, C1QBP
HNRNPA2B1
CYBA, TXN
CLIC1, SYNE2
PARK7, CALR
GNAS
IL7R
EEF1A1
ATP1A1
RPSA
FABP5
2
1
0
−1
−2
Expression
I
Up-regulated
Down-regulated
nuclear-transcribed mRNA catabolic process
ribonucleoprotein complex subunit organization
antigen processing and presentation of peptide antigen
Fc receptor signaling pathway
response to interleukin-12
-log10(P-value)
0
7
J
IL7R
IL2
Expression
4
3
2
1
0
2
1
0
COVID-19
HC
